# Comparison of treatment response, remission rate and drug adherence in polyarticular juvenile idiopathic arthritis patients treated with etanercept, adalimumab or tocilizumab

**DOI:** 10.1186/s13075-016-1170-3

**Published:** 2016-11-24

**Authors:** Gerd Horneff, Ariane Klein, Jens Klotsche, Kirsten Minden, Hans-Iko Huppertz, Frank Weller-Heinemann, Jasmin Kuemmerle-Deschner, Johannes-Peter Haas, Anton Hospach

**Affiliations:** 1 Department of Pediatrics, Centre of General Pediatrics and Neonatology, Asklepios Clinic Sankt Augustin, 53757 Sankt Augustin, Germany; 2 German Rheumatism Research Centre Berlin, and Children’s University Hospital Charité, Berlin, Germany; 3 Prof Hess Children’s Hospital, Bremen, Germany; 4 Department of Pediatrics, University of Tuebingen, 72076 Tuebingen, Germany; 5 German Centre for Pediatric and Adolescent Rheumatology, Garmisch-Partenkirchen, Germany; 6 Department of Pediatrics, Section of Rheumatology, Olgahospital, Klinikum, Stuttgart, Germany

**Keywords:** Adalimumab, Etanercept, Tocilizumab, Juvenile idiopathic arthritis, Drug surveillance, JADAS

## Abstract

**Background:**

Treatment response, remission rates and compliance in patients with polyarticular juvenile idiopathic arthritis (polyJIA) treated with adalimumab, etanercept, or tocilizumab were analyzed in clinical practice.

**Methods:**

Data collected in the German BIKER registry were analyzed in patients with polyJIA who started treatment with approved biologics, adalimumab, etanercept or tocilizumab, from 2011 to 2015. Baseline patient characteristics, treatment response, safety and drug survival were compared.

**Results:**

Two hundred thirty-six patient started adalimumab, 419 etanercept and 74 tocilizumab, with differences in baseline patient characteristics. Baseline Juvenile Disease Activity Score (JADAS)10 (mean ± SD) in the adalimumab/etanercept/tocilizumab cohorts was 12.1+/−7.6, 13.8 ± 7.1 and 15.1 ± 7.4, respectively (adalimumab vs etanercept, *p* = 0.01), and Childhood Health Assessment Questionnaire (CHAQ)-disability index scores was 0.43 ± 0.58, 0.59 ± 0.6 and 0.63 ± 0.55, respectively (adalimumab vs etanercept, *p* < 0.001). Uveitis history was more frequent in the adalimumab cohort (OR 5.73; *p* < 0.001). Balanced patients’ samples were obtained by a generalized propensity score to adjust for baseline differences. Pediatric ACR30/50/70/90 criterion improvement after 3 months treatment was achieved by 68%/60%/42%/24% in the etanercept cohort, 67%/59%/43%/27% in the adalimumab cohort and 61%/52%/35%/26% in the tocilizumab cohort. At 24 months, JADAS minimal disease activity was achieved in 52.4%/61.3%/52.4% and JADAS remission in 27.9%/34.8%/27.9% patients in the adalimumab/etanercept/tocilizumab cohorts, respectively. Etanercept was used in 95.5% of patients as a first biologic, adalimumab in 50.8% and tocilizumab in 20.2%. There were no important differences in efficacy between first-line and second-line use of biologics. In total 60.4%/49.4%/31.1% patients discontinued adalimumab/etanercept/tocilizumab, respectively (HR for adalimumab 1.67; *p* < 0.001; HR for tocilizumab 0.35; *p* = 0.001). Drug survival rates did not differ significantly in patients on biologic monotherapy compared with combination therapy with methotrexate. Over 4 years observation under etanercept/adalimumab/tocilizumab, 996/386/103 adverse events, and 148/119/26 serious adverse events, respectively, were reported.

**Conclusions:**

In clinical practice, etanercept is most frequently used as first-line biologic. Adalimumab/etanercept/tocilizumab showed comparable efficacy toward polyJIA. Overall, tolerance was acceptable. Interestingly, compliance was highest with tocilizumab and lowest with adalimumab. This study provides the first indication for the comparison of different biologic agents in polyarticular JIA based on observational study data with all their weaknesses and demonstrates the need for well-controlled head-to-head studies for confirmation.

**Electronic supplementary material:**

The online version of this article (doi:10.1186/s13075-016-1170-3) contains supplementary material, which is available to authorized users.

## Background

Juvenile idiopathic arthritis (JIA) is a collective term for arthritides that are diagnosed before the age of 16 years. Diagnosis requires disease duration of at least 6 weeks and the exclusion of other causes of arthritis [1]. JIA is the most common chronic rheumatic inflammatory disease of childhood. If not successfully treated, it can lead to severe disability [2].

Pharmacologic treatment consists of nonsteroidal antirheumatic drugs, mainly for symptomatic relief, and disease-modifying antirheumatic drugs (DMARDs). Of the latter group, methotrexate (MTX) is the most common first-line DMARD and is a cornerstone drug in the treatment of patients with JIA. Its efficacy was first demonstrated two decades ago in a randomized controlled trial [3]. According to national and international guidelines and recommendations, patients with JIA who are refractory to MTX treatment are eligible for treatment with biologic agents [4, 5].

Etanercept, an anti-TNF-α receptor immunoglobulin Fc fragment fusion protein, was the first biologic agent approved by the US Food and Drug Administration for the treatment of polyarticular JIA (pJIA) in 1999 and by the European Medicines Evaluating Agency in 2000. Its efficacy and safety were demonstrated in a randomized controlled withdrawal trial and several long-term observational studies from national registries, including the German Biologics in Pediatric Rheumatology (BIKER) Registry [6–9]. In 2008, adalimumab, a monoclonal anti-TNF-α antibody, was approved for the treatment of polyarticular JIA, as monotherapy or in combination with MTX, after its efficacy was established in a placebo-controlled withdrawal trial [10]. Adalimumab was preferred over etanercept for the treatment of uveitis for years until a recent randomized placebo controlled trial demonstrated its efficacy for treatment of uveitis [11]. Observational data on the use of adalimumab for JIA are more limited than those on the use of etanercept [12]. Tocilizumab, a monoclonal interleukin-6 receptor antibody, was first approved for systemic onset JIA in 2011 and was found to be effective in a randomized controlled withdrawal trial [13]. Since 2013, Tocilizumab has been the third first-line biologic agent approved for treatment of polyarticular JIA.

The efficacy of these three agents for the treatment of JIA is considered to be equivalent [14]. However, no head-to-head trials have been conducted to compare etanercept, adalimumab or tocilizumab. All three biologic agents are approved for treatment of polyarticular JIA in children older than 2 years who do not respond to MTX. Therefore, the initial decision of which biologic agent to use must be determined based on limited evidence. The aim of this analysis was to compare baseline characteristics, efficacy, tolerability and drug survival in patients with polyarticular JIA initiating adalimumab, etanercept or tocilizumab.

## Methods

The German BIKER Registry was approved by the local ethics committee. Written consent was obtained and pseudonymized data were collected. This registry has been extensively described in previous reports [9, 15]. Patients in the German BIKER registry initiating treatment with adalimumab, etanercept or tocilizumab between 1 January 2011 and 31 December 2015 were included in the study. The study population was restricted to patients who were classified in the following JIA categories: rheumatoid factor-positive polyarthritis, rheumatoid factor-negative polyarthritis and extended oligoarthritis. Patients with other JIA categories (systemic onset JIA, persistent oligoarthritis, psoriatic arthritis (PsA), enthesitis-related arthritis (ERA) and unclassified JIA) were excluded because of differences in the approval of the three biologics studied and to homogenize the study population.

For the efficacy analyses, assessments were performed at baseline and at follow up after 3 and 6 months and every 6 months thereafter. Due to the character of the registry study, the number of patients observed decreased with treatment duration. For this reason, efficacy was analyzed until month 24. Juvenile Disease Activity Score (JADAS) scores and improvement in the Pediatric American College of Rheumatology Criteria (PedACR) were calculated as previously described in detail [16, 17]. The JADAS minimal disease activity (MDA) (defined as JADAS10 ≤ 3.8) and JADAS remission rates (defined as JADAS10 ≤ 1) according to the definition of Consolaro et al. [18] were calculated. The JADAS10 was chosen because all four domains, number of active joints (truncated at 10), patient’s/parent’s global assessment of disease activity, physician’s global assessment of disease activity, and erythrocyte sedimentation rate, ranged from 0 to 10 points. Functional status was determined using the Childhood Health Assessment Questionnaire (CHAQ) disability index [19].

Safety was analyzed based on adverse event reporting. An adverse event was defined as any untoward medical occurrence in a subject administered a pharmaceutical product, even without a causal relationship with the treatment. Serious adverse events included death, a life-threatening event or an event leading to or prolonging hospitalization, persistent or significant disability/incapacity or an important medical event requiring medical or surgical intervention to prevent a serious outcome or congenital anomaly or birth defect. For this analysis, reasons for discontinuation were classified as inefficacy, intolerance, remission or other reasons.

### Statistical analysis

The adalimumab, etanercept and tocilizumab cohorts differed in their clinical characteristics and treatment history at baseline. A generalized propensity score was estimated to obtain balanced samples of patients in respect to baseline characteristics. The likelihood of being allocated to a cohort was estimated by a multinomial logistic regression model, including the predictors, sex, age at JIA onset, JIA category, disease duration, JADAS10, concomitant MTX use and the number of previously used biologic agents. Balanced samples of patients were obtained using an inverse probability of treatment weight. Generalised estimation equations (GEE) were applied to the weighted sample of patients to analyze the drug adherence and treatment response. Drug survival was analyzed by Kaplan-Meier plots and Cox proportional hazard model.

## Results

### Study population

The German BIKER registry database of 3547 patients with JIA was used to identify eligible patients. Patients diagnosed with rheumatoid factor-positive polyarthritis, rheumatoid factor-negative polyarthritis or extended oligoarthritis, who had initiated treatment with a biologic agent from 1 January 2011 to 31 December 2015, were considered. Up to December 2015, 236 patients started on adalimumab, 419 started on etanercept and 74 started on tocilizumab.

Clinical characteristics at treatment initiation significantly differed between the three groups (Additional file 1: Table S1). The propensity-score-weighted analyses of baseline characteristics resulted in balanced samples as reported in Table 1 except for sex and uveitis. Female predominance was more pronounced in the etanercept and adalimumab cohorts than in the tocilizumab cohort (Table 1). Compared with the etanercept cohort, patients in the adalimumab or tocilizumab cohort were slightly older. The distribution of the three analyzed JIA categories was comparable among medications despite a lower rate of patients diagnosed with extended oligoarthritis in the tocilizumab cohort compared with the other two cohorts combined.

**Table 1:** Patients’ characteristics (absolute number of patients, percentages, descriptive statistics as reported in BIKER; comparison between the three cohorts weighted by an inverse probability of treatment)

	Etanercept cohort	Adalimumab cohort	Tocilizumab cohort	Etanercept versus adalimumab^a^	Tocilizumab versus etanercept^a^	Tocilizumab versus adalimumab^a^
	*n* = 419	*n* = 236	*n* = 74	OR (95% CI); *p* value	OR (95% CI); *p* value	OR (95% CI); *p* value
Female, *n* (%)	332 (79.2%)	192 (81.4%)	51 (68.8%)	0.96 (0.57; 1.62); 0.88	0.63 (0.45; 0.89); 0.03	0.58 (0.48; 0.98); 0.04
Age at baseline, years, mean ± SD	10.5 ± 4.4	11.8 ± 4.0	12.9 ± 3.6	0.63 (−0.31; 1.57); 0.19	1.65 (−0.67; 3.96); 0.16	1.02 (−1.31; 3.34); 0.39
Median (IQR)	11.1 (7.1–13.9)	12.7 (8.7– 15.0)	13.5 (11.2– 15.9)			
Disease duration at treatment start, mean ± SD	3.6 ± 3.3	5.8 ± 4.0	6.1 ± 3.5	0.40 (−0.17; 0.98); 0.17	1.13 (−0.05; 2.02); 0.07	0.73 (−0.21; 1.66); 0.13
Median (IQR)	2.6 (1.1–5.1)	4.9 (2.4–8.4)	5.8 (2.9–8.8)			
JIA category *n* (%)						
RF+ PA	37 (8.8%)	23 (9.7%)	9 (12.2%)	1.45 (0.74; 2.83); 0.28	0.95 (0.60; 1.49); 0.81	2.18 (0.48; 9.85); 0.31
RF- PA	224 (53.5%)	128 (54.2%)	47 (63.5%)	(ref)	(ref)	(ref)
ExOA	158 (37.7%)	85 (36.0%)	18 (24.3%)	3.17 (0.74; 13.60); 0.12	0.71 (0.22; 2.27); 0.57	0.75 (0.24; 2.41); 0.63
First biologic used	400 (95.5%)	110 (46.6%)	14 (18.9%)	0.54 (0.28; 1.03); 0.06	0.44 (0.16; 1.18); 0.10	0.81 (0.34; 1.96); 0.65
Co-med corticosteroids, *n* (%)	134 (32.0)	60 (25.4)	26 (35.1)	1.38 (0.96; 1.97)	1.15 (0.69; 1.94)	1.59 (091; 2.78)
Co-med MTX, *n* (%)	302 (72.1)	127 (53.8)	34 (45.9)	1.20 (0.76; 1.88); 0.44	0.76 (0.28; 2.06); 0.59	0.64 (0.24; 1.70); 0.37
JADAS10 (0–40), mean ± SD	13.8 ± 7.1	12.1 ± 7.6	15.1 ± 7.4	−0.41 (−2.30; 1.48); 0.67	−0.53 (−4.22; 3.17); 0.78	−0.12 (−3.84; 3.60); 0.95
Median (IQR)	13.6 (8.8–19.0)	11.7 (6.1–17.5)	14.8 (9.2–20.1)			
CHAQ-DI (0–3), mean ± SD	0.59 ± 0.60	0.43 ± 0.58	0.63 ± 0.55	−0.04 (−0.19; 0.12); 0.64	−0.10 (−0.30; 0.11); 0.35	−0.06 (−0.29; 0.17); 0.60
Median (IQR)	0.38 (0.13–0.88)	0.13 (0–0.623)	0.63 (0.19-1.0)			
Uveitis before start of biologic	23 (5.5%)	59 (25%)	0	3.41 (3.21; 4.45); 0.03	-	-

^a^Analyses weighted by an inverse probability of treatment estimated by a generalized propensity score. *beta* regression coefficient for continuous variables, *CI* confidence interval, *OR* odds ratio for categorical variable, *(ref)* reference group, *JIA* juvenile idiopathic arthritis, *RF* rheumatoid factor, PA, polyarthritis, ExOA extended oligoarthritis, *JADAS* Juvenile Disease Activity Score, *CHAQ-DI* Childhood Health Assessment Questionnaire disability index

Etanercept was used as a first biologic agent in 583 patients (79.9%), adalimumab in 174 patients (23.9%) and tocilizumab in 17 patients (2.3%). Thus, etanercept was preferred as a first-line biologic agent. Only 17 patients (4.1%) received etanercept as a second-line biologic agent. In the adalimumab cohort, approximately half (46.6%) of the patients received adalimumab as the first biologic agent, while only 18.9% in the tocilizumab cohort received tocilizumab as the first biologic agent.

Compared with the etanercept cohort, the baseline JADAS10 and CHAQ-disability index were lower in the adalimumab cohort. Concomitant uveitis was present in 23 (5.5%) of the etanercept cohort, in 58 (25%) of the adalimumab cohort and in no patients in the tocilizumab cohort. Thus, uveitis demonstrated the highest frequency in patients treated with adalimumab (odds ratio 3.41 (95% CI 3.21; 4.45); *p* = 0.03).

At baseline, 302 patients (72%) in the etanercept cohort but only 127 (54%) in the adalimumab and 34 (46%) in the tocilizumab cohort received concomitant therapy with MTX. The differences described were not statistically significant after weighting the analyses with the propensity score weight (Table 1).

### Treatment response

**Fig. 1:**
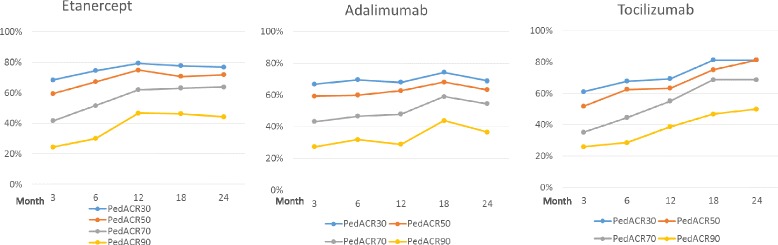
Improvement in patients using etanercept, adalimumab or tocilizumab according to the Pediatric American College of Rheumatology (*PedACR*)30/50/70 and 90 criteria

The mean treatment duration was comparable among all cohorts (1.25 ± 1.05 years in the etanercept cohort, 1.0 ± 0.86 in the adalimumab cohort and 0.98 ± 0.59 in the tocilizumab cohort). Improvement according to PedACR30/50/70/90 criteria was reached after 3 months by 68%/60%/42 %/24 % in the etanercept cohort, 67%/59%/43%/27% in the adalimumab cohort and 61%/52%/35%/26% in the tocilizumab cohort, respectively. The response rates further increased or were stable with continuing treatment (Fig. 1). There were no statistically significant differences between the three groups in the PedACR response rates. PedACR response rates to etanercept/adalimumab/tocilizumab, either as first-line or second-line biologic agents, were comparable (Additional file 2: Figure S1).

**Fig. 2:**
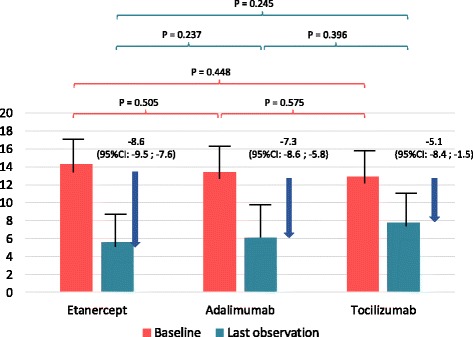
Improvement in patients following etanercept, adalimumab or tocilizumab treatment according to Juvenile Disease Activity Score (*JADAS*)10 at baseline compared with the last observation on a study drug

At baseline, the mean observed JADAS10 was highest in the tocilizumab (15.1 ± 7.4) and etanercept (13.8 ± 7.1) cohorts (*p* > 0.05) and significantly lower in the adalimumab cohort (12.1 ± 7.6; *p* = 0.003 compared to etanercept; *p* = 0.011 compared to tocilizumab). The significant differences in baseline JADAS10 disappeared after weighting the analyses (Table 1, Fig. 2).

A significant decrease in the adjusted JADAS10 was observed in all three cohorts (Fig. 2). From baseline to the last observation on treatment, the JADAS10 decreased by 8.6 (95% CI 7.6; 9.5) points in the etanercept cohort, by 7.3 (95% CI 5.8; 8.6) in the adalimumab cohort and by 5.1 (95% CI 1.5; 8.4) in the tocilizumab cohort. The decrease in JADAS10 did not significantly differ among the three cohorts. The mean decrease in JADAS10 was −7.7 (95% CI −8.47; −6.99) in patients with rheumatoid factor-negative polyarthritis or extended oligoarthritis, and −9.4 (95% CI −12.13; −6.63) for rheumatoid factor-positive polyarthritis. The difference between the JIA categories in the JADAS10 response was not statistically significant (delta = 1.51; 95% CI −0.54; 3.56; *p* = 0.149).

**Fig. 3:**
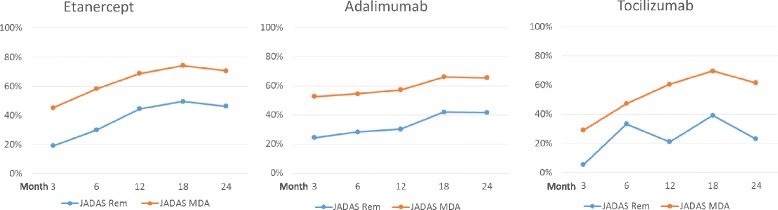
Rates of Juvenile Disease Activity Score (*JADAS*)10 remission and minimal disease activity in patients taking etanercept, adalimumab or tocilizumab

JADAS remission and JADAS-MDA were used as further treatment response indicators. In the etanercept, adalimumab and tocilizumab cohorts, 131 patients (34.8%), 71 patients (27.9%) and 16 patients (23.5%), respectively, achieved JADAS remission (defined as JADAS10 ≤ 1.0) at the last observation. The three cohorts did not significantly differ in JADAS remission at the last observation adjusting for baseline differences between the three cohorts. The rates of achieving JADAS-MDA were comparable among all three cohorts (Fig. 3): 231 patients (61.3%), 133 patients (52.4%) and 32 patients (47.1%) in the etanercept, adalimumab and tocilizumab cohorts, respectively, achieved JADAS-MDA (defined as JADAS10 ≤ 3.8) at the last observation.

Patients who were first exposed to biologic agents were compared to those who switched from a first to a second biologic agent. The mean (± SD) JADAS10 in the cohort previously exposed to a biologic agent (14.8 ± 7.3) was not significantly lower than that in the biologic-agent-naïve cohort (16.5 ± 7.2). The decline in JADAS10 at 3, 6, 12, 18 and 24 months, respectively, was 3.9 ± 3.3, 6.2 ± 7.4, 9.6 ± 7.7, 6.6 ± 5.9 and 2.9 ± 2.4 in the biologic-naïve group, and 8.9 ± 6.5, 7.6 ± 8.2, 4.6 ± 5.6, 3.2 ± 4.5 and 7.7 ± 8.8 in the cohort switching from a first to a second biologic. The decline in JADAS and the rates of achieving JADAS remission and JADAS- MDA were again comparable among the cohorts.

Functional disability was analyzed using the CHAQ-DI. The observed baseline CHAQ-DI was highest in the tocilizumab cohort (0.63 ± 0.55), followed by the etanercept cohort (0.59 ± 0.60) and the adalimumab cohort (0.43 ± 0.58). However, the baseline CHAQ-DI did not differ among the three cohorts in the propensity-score-weighted analyses. Reductions in CHAQ-DI from baseline to the last observation in the tocilizumab, etanercept and adalimumab cohorts were (−0.31 ± 0.46), (−0.22 ± 0.54) and (−0.12 ± 0.47), respectively. Reduction in the CHAQ-DI was significantly greater in the etanercept cohort than in the adalimumab cohort (*p* = 0.032). There was no significant difference in the reduction in the CHAQ-DI between the tocilizumab and the etanercept cohort (*p* = 0.261). However, the residual CHAQ-DI at the last observation was comparable among all three cohorts (etanercept 0.31 ± 0.44, adalimumab 0.29 ± 0.48 and tocilizumab 0.36 ± 0.46).

### Drug adherence and discontinuations

**Fig. 4:**
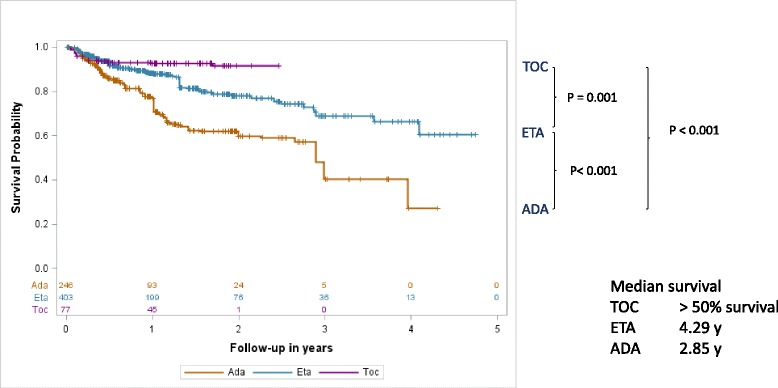
Drug survival during treatment with etanercept (ETA), adalimumab (*ADA*) or tocilizumab (*TOC*); weighted Kaplan-Meier analyses weighted by an inverse probability of treatment estimated by a generalized propensity score. Significant differences were noted, using the Cox proportional hazard model, between the cohorts treated with adalimumab versus etanercept (*p* < 0.001, hazard ratio 0.60 (0.48–0.75)), adalimumab versus tocilizumab (*p* = 0.001, hazard ratio 0.21 (0.12 − 0.39)) and tocilizumab versus etanercept (*p* < 0.001, hazard ratio 2.82 (1.55 − 5.14))

Drugs were discontinued by 142 patients (60.2%) in the adalimumab cohort versus 207 patients (49.4%) in the etanercept cohort and 23 patients (31.1%) in the tocilizumab cohort. The median drug survival before discontinuation due to inefficacy or intolerance was 2.85 years for adalimumab and 4.29 years for etanercept; this was not calculated for tocilizumab because more than 50% of the patients were still receiving treatment (Fig. 4). Survival analyses using Cox proportional hazard regression revealed significant differences between adalimumab and etanercept survival (hazard ratio 2.82, 95% CI 1.55; 5.14; *p* < 0.001), between adalimumab and tocilizumab survival (hazard ratio 4.71, 95% CI 2.58; 8.61; *p* < 0.001), and between etanercept and tocilizumab survival (hazard ratio 2.82, 95% CI 1.55; 5.14; *p* = 0.001).

Drug survival with adalimumab (hazard ratio 1.36, 95% CI 1.03; 1.78; *p* = 0.029) was improved but was significant lower compared to etanercept when restricting the analyses to patients who took etanercept and adalimumab as their first biologic agents. Interestingly, the survival rates of patients on monotherapy with a biologic agent compared with combination therapy with MTX were not significantly different (*p* = 0.836) regardless of whether they were calculated separately in the etanercept, adalimumab or tocilizumab cohort (data not shown) or for the combined analysis (Additional file 3: Figure S2). Patients with rheumatoid factor-negative polyarthritis or extended oligoarthritis had a comparable drug survival to patients with rheumatoid factor-positive polyarthritis (hazard ratio = 0.995, 95% CI 0.67; 1.47; *p* = 0.98). Drug survival also did not statistically significantly differ between the JIA categories in the adalimumab (hazard ratio = 1.15, 95% CI 0.62; 2.12; *p* = 0.653) and etanercept (hazard ratio = 0.83, 95% CI 0.48; 1.43; *p* = 0.502) cohorts.

The reasons for discontinuation are provided in Table 2. The most common reason for discontinuation was poor efficacy or unsatisfactory response. Significantly more patients discontinued adalimumab (odds ratio 4.89, 95% CI 1.65; 14.48; *p* = 0.004) for reasons of inefficacy (*n* = 52 (22%)) than tocilizumab (*n* = 9 (12.2%)). Remission was the second leading reason for discontinuation of a biologic agent. Significantly more patients discontinued etanercept due to remission (*n* = 54 (12.9%; odds ratio 8.24 (95% CI 1.27; 53.53); *p* = 0.027) than tocilizumab (*n* = 2 (2.7%)). In general, few patients discontinued due to intolerance (etanercept, *n* = 15 (3.6%); adalimumab, *n* = 15 (6.4%); tocilizumab, *n* = 2 (2.7%)). Patients treated with adalimumab had a higher odds for discontinuation due to intolerance (odds ratio 2.28, 95% CI 1.03; 5.04; *p* = 0.042) than etanercept. The reasons for discontinuation included infections, hypersensitivity, neuropsychiatric events, vasculitis, lymphoma (one patient) and others (Table 2).

**Table 2:** Rates and reasons for discontinuation

	Etanercept cohort	Adalimumab cohort	Tocilizumab cohort	Adalimumab versus etanercept^a^	Tocilizumab versus etanercept^a^	Tocilizumab versus adalimumab^a^
	*n* = 419	*n* = 236	*n* = 74	OR (95% CI); *p* value	OR (95% CI); *p* value	OR (95% CI); *p* value
Discontinuations, *n* (%)	207 (49.4)	142 (60.4)	23 (31.1)	1.57 (1.03; 2.41); 0.037	0.20 (0.09; 0.45); <0.001	0.13 (0.06; 0.29); <0.001
Inefficacy, *n* (%)	50 (11.9)	52 (22.0)	9 (12.2)	1.65 (0.88; 3.08); 0.118	0.34 (0.11; 1.00); 0.050	0.20 (0.07; 0.60); 0.004
Remission, *n* (%)	54 (12.9)	22 (9.3)	2 (2.7)	0.78 (0.43; 1.40); 0.404	0.12 (0.02; 0.79); 0.027	0.16 (0.02; 1.05); 0.056
Intolerance, *n* (%)	15 (3.6)	15 (6.4)	2 (2.7)	2.28 (1.03; 5.04); 0.042	0.84 (0.18; 4.01); 0.826	0.37 (0.08; 1.79); 0.216
Details	Hypersensitivity (5), uveitis (3), vasculitis (1),	Infections (4)^b^	Impetigo (1)			
	Lymphoma (1)	Hypersensitivity (3)	Neutropenia (1)			
	Elevated transaminases (1), neuro-psychiatric (4)^b^	Pustulosis (1), neuro-psychiatric (5)^b^		0.2	5	
Others*, *n* (%)	88 (16.0)	53 (22.4)	10 (13.4)	1.21 (0.74; 1.96); 0.443	0.27 (0.10; 0.72); 0.009	0.22 (0.08; 0.60); 0.003

^a^Analyses weighted by an inverse probability of treatment estimated by a generalized propensity score. ^b^Infections included pneumonia and soft tissue infections; Neuropsychiatric included headache, nausea, aggressiveness, anxiety, and vertigo. *beta* regression coefficient for continuous variables, *CI* confidence interval, *OR* odds ratio for categorical variable

### Safety

A total of 1484 adverse events (AE) were reported, of which there were 996 in patients receiving etanercept, 386 in patients receiving adalimumab and 102 in patients receiving tocilizumab. Of the adverse events, 148 were classified as serious adverse events (SAE), comprising 119 patients on etanercept, 26 patients on adalimumab and 3 patients on tocilizumab. Thus, the rates of AE and SAE were significantly higher with etanercept than with the other biologic agents (Table 3).

**Table 3:** Safety

	ADA	ETA	TOC	ETA vs ADA	ADA vs TOC	ETA vs TOC
Patients, *n*	236; 236.4	419	74			
Patient years, *n*	236.4	524,096	72,47364819			
Exposure, years mean ± SD	1.00 ± 0.86	1.25 ± 1.05	0.98 ± 0.60			
Adverse events, *n*; *n*/patient	386; 1.63	996; 2.37	102: 1.38	*p* = 0.011		*p* = 0.004
Rate/100 PY (95% CI)	163.3 (148.8; 180.4)	190.0 (178.6; 202.2)	140,7 (113.5; 167.4)	RR 1.16 (1.03–1.31)	ns	RR 1.35 (1.1–1.66)
Serious adverse events, *n*; *n*/patient	26; 0.11	119; 0.28	3; 0.04	*p* = 0.0008		*p* = 0.004
Rate/100 PY (95% CI)	11.0 (7.5; 16.2)	22.07 (19.0; 27.2)	4.1 (1.3; 12.8)	RR 2.06 (1.35–3.16)	ns	RR 5.48 (1.74–17.25)
Autoimmunopathy, *n*; *n*/patient	3; 0.012	2; 0.004	1; 0.014			
Rate/100 PY (95% CI)	1.27 (0.41; 3.39)	0.38 (0.09; 1.53)	1.38 (0.19; 9.59)	ns	ns	ns
Bleeding disorder, n; *n*/patient	2; 0.008	3; 0.007	0			
Rate/100 PY (95% CI)	0.85 (0.21; 3.38)	0.57 (0.18; 1.17)		ns	ns	ns
CED, n; *n*/patient	1; 0.004	13; 0.031	0	*p* = 0.09		
Rate/100 PY (95% CI)	0.42 (0.06; 3.0)	2.48 (1.44; 4.27)		RR 5.86 (0.77–44.83)	ns	ns
Demyelinisation	0	1; 0.002	0			
Rate/100 PY (95% CI)		0.19 (0.03-1.35)		ns	ns	ns
Hepatitis	6; 0.025	10; 0.024	3; 0.041			
Rate/100 PY (95% CI)	2.54 (1.14; 5.65)	1.91 (1.03; 3.55)	4.14 (1.31; 12.57)	ns	ns	ns
Hyperlipidemia	0	3; 0.007	1; 0.014			
Rate/100 PY (95% CI)		0.57 (0.18; 1.17)	1.38 (0.19; 9.59)	ns	ns	ns
Infection. serious or medically important; *n*; *n*/patient	13; 0.055	50; 0.119	3; 0.041	*p* = 0.076		
Rate/100 PY (95% CI)	5.5 (3.19; 9.47)	9.54 (7.23; 12.59)	4.14 (1.31; 12.57)	RR 1.73 (0.94–3.19)	ns	ns
Intolerance, n; *n*/patient	2; 0.008	3; 0.007	2; 0.027			
Rate/100 PY (95% CI)	0.85 (0.21; 3.38)	0.57 (0.18; 1.17)	2.76 (0.68; 10.81)	ns	ns	ns
Malignancy, n; *n*/patient	0	1; 0.002	0			
Rate/100 PY (95% CI)		0.19 (0.03; 1.35)		ns	ns	ns

*ADA* adalimumab, *ETA* etanercept, *TOC* tocilimuzab, *PY* person years, *ns* not significant, *RR* relative risk

The AE and SAE were classified as adverse events of special interest (AESI) if they represented new onset or aggravation of uveitis (*n* = 88), serious or medically important infections (*n* = 66), neutropenia (*n* = 26), hepatitis/elevated transaminases (*n* = 19), chronic inflammatory bowel disease (CED, *n* = 14), intolerance (*n* = 7), new onset of autoimmunity (*n* = 6), pregnancy (*n* = 6), bleeding disorders (*n* = 5), hyperlipidemia (*n* = 4), stroke (*n* = 2), malignancy (*n* = 1), thrombosis (*n* = 1), or demyelination (*n* = 1). There were no deaths.

Serious or medically important infections observed in the etanercept cohort included pneumonia (*n* = 9), primary varicella (*n* = 7, none were vaccinated), zoster (*n* = 14), pyelonephritis (*n* = 5), peritonitis (*n* = 2), appendicitis (*n* = 2) and cellulitis (*n* = 2). The events observed with adalimumab were pneumonia (*n* = 2), primary varicella (*n* = 4, none were vaccinated), zoster (*n* = 3), osteomyelitis (*n* = 1), septic arthritis [1] appendicitis (*n* = 1) and influenza (*n* = 1). For tocilizumab, one case each of pneumonia, appendicitis and influenza was reported. No cases of tuberculosis occurred, and apart from herpes zoster, no opportunistic infections were observed.

Some differences in the occurrence of specific AESI were noted among the three treatment cohorts. Neutropenia and serious or medically important infections occurred with significantly greater frequency in the etanercept cohort compared with the adalimumab cohort. Aggravation of uveitis occurred more frequently in the adalimumab cohort than in the tocilizumab cohort, whereas neutropenia occurred significantly less frequently. Compared with the tocilizumab cohort, aggravation and new onset of uveitis occurred more frequently in the etanercept cohort.

Notably, serious or medically important infections and events of chronic inflammatory bowel disease occurred more frequently in the etanercept cohort than in the adalimumab cohort. However, this difference was not significant (*p* < 0.05, Table 3).

## Discussion

To our knowledge, this is the first study to directly compare adalimumab, etanercept, and tocilizumab as therapy for polyarticular JIA. Data analysis was restricted to patients initiating treatment after 2011, when tocilizumab became available for the treatment of JIA. The patient population was further restricted to patients with rheumatoid factor-positive or rheumatoid factor-negative polyarthritis and extended oligoarthritis. Patients with systemic JIA (sJIA), enthesitis-related arthritis (ERA) and psoriatic arthritis (PsA) were excluded to acquire a more homogenous patient population and to avoid the effect of differences in approval among the respective drugs; etanercept is approved for ERA and PsA, adalimumab for ERA and tocilizumab for sJIA.

Etanercept was approved for polyarticular JIA in 2000, and adalimumab was approved in 2008. Therefore, considerably more experience has been gained with etanercept than with adalimumab or tocilizumab. Accordingly, etanercept was used much more frequently than either adalimumab or tocilizumab. In the Dutch ABC registry, etanercept was also the most frequently prescribed biologic agent for non-systemic JIA [20]. In that study, greater drug experience was the most important factor driving the decision to use etanercept rather than adalimumab. Adalimumab was favored by Dutch pediatric rheumatologists for patients who had preceding uveitis. Similar findings were obtained in the current BIKER population.

For German pediatric rheumatologists, we may speculate that the number of patients starting on etanercept as their first biologic agent is higher because there is prolonged experience with etanercept compared to the other biologic agents. Adalimumab was predominantly chosen as the first biologic agent in patients with concomitant uveitis. Finally, the relative rarity of using tocilizumab can either be attributed to the fact that first, it is the biologic agent with the least experience of use and second, that currently only the intravenous infusion is approved, which means it can only be used in centers with an infusion facility. The decision as to which biologic agent is used is not influenced by a protocol, algorithm, national recommendation or guideline, or by the registry itself. Adalimumab was used more frequently than etanercept as a second-line biologic agent but had comparable efficacy in both second-line and first-line users [12].

There were few other differences among the studied cohorts. Patients in the adalimumab or tocilizumab cohorts were older than those in the etanercept cohort, and there were fewer patients with extended oligoarthritis in the tocilizumab cohort. A striking difference was observed in use of concomitant MTX, which was much more common in the etanercept cohort, followed by the adalimumab cohort. Fewer than 50% of the patients in the tocilizumab cohort received concomitant MTX therapy. This observation was surprising because etanercept is approved only for monotherapy, whereas both of the other biologic agents are also approved for combination therapy.

Despite these differences, PedACR treatment response rates were comparable for all three biologic agents. The majority of patients demonstrated marked improvement after 3 months of treatment. The response rates thereafter remained stable or increased further. No apparent differences among the three treatment cohorts were noted, in accordance with the response rates observed in open-label phases of the pivotal randomized controlled withdrawal studies of each of the drugs [6, 13, 21]. However, in the most recent clinical trial investigating etanercept, patients with extended oligoarticular JIA experienced a much higher rate of clinical improvement [9].

There was a small but significant difference in reported absolute disease activity at baseline, as measured by the JADAS10, which was highest in the tocilizumab cohort and lowest in the adalimumab cohort. This difference was balanced by the inverse probability of treatment allocation estimated by a generalized propensity score. The JADAS score fell significantly in all cohorts after treatment. The residual JADAS at the last observation (mean observation time in all cohorts of approximately 1 year) was highest in the etanercept cohort. The greatest adjusted mean reduction in the JADAS was observed in the etanercept cohort; in contrast, the greatest observed mean reduction was in the tocilizumab cohort.

As tocilizumab may have had a higher influence on the acute phase response via its inhibitory capability on the IL-6 axis, the JADAS10 and the clinical JADAS (cJADAS), which considers only three clinical parameters were compared. Interestingly, at baseline 144 of 407 patients (35.4%) in the etanercept cohort and 26 of 74 patients (35.1%) in the tocilizumab cohort had higher classic JADAS than cJADAS. Upon treatment (month 3 to 24), 91 of 856 patients (10.6%) in the etanercept cohort and 22 of 187 patients (12.4%) from the tocilizumab cohort had higher classic JADAS than cJADAS. Thus, both at baseline and after treatment the results were very much comparable, and direct inhibition of IL-6 seems not to influence the laboratory parameters of the JADAS10 more than TNF inhibition. As we used definitions for MDA and remission based on the complete JADAS, the original JADAS10 was preferred over the cJADAS, which considers only three clinical parameters. Comparable numbers of patients achieved JADAS remission and JADAS minimal disease activity in a large database study [16, 18]. No differences were identified among the cohorts in terms of either speed to remission/MDA or absolute remission/MDA rates.

Interestingly, the drug survival rates for the three biologic agents were different in this study. Whereas etanercept and tocilizumab showed comparable drug survival, significantly more patients discontinued adalimumab. The main reason for adalimumab discontinuation was lack of therapeutic response or inefficacy, which differed significantly compared with the discontinuation frequency with etanercept due to inefficacy/lack of therapeutic effect. This could have been influenced by the higher proportion of patients on MTX in the etanercept cohort. It is possible that Adalimumab in monotherapy could lead to more immunogenicity and secondary loss of efficacy.

Remission was the second leading reason for drug discontinuation. Significantly fewer patients discontinued tocilizumab than etanercept for reasons of remission. This finding is remarkable because the rates of JADAS remission were not different. The mean/median duration of treatment with both etanercept and tocilizumab were comparable. There were also no differences in the number of patients on prolonged treatment: a quarter of the patients had been treated with etanercept for more than 1.7 years, which was comparable to more than 1.5 years in a quarter of the patients using tocilizumab. It is likely that tocilizumab adherence was affected by its more frequent use as a second-line biologic agent compared with etanercept and thus an earlier biologic treatment had failed. It can be speculated that in this situation, successful drug treatment likely will not be discontinued.

Very few patients discontinued their biologic agent due to intolerance, which suggests that they are not only very effective but also very well-tolerated. The pattern of AE observed in this study of patients with pJIA is consistent with the known safety profile of adalimumab, etanercept and tocilizumab [6, 10, 13]. Interestingly, there were no cases of tuberculosis. Apart from herpes zoster, which has been regarded as an opportunistic infection by some authors, no other opportunistic infections were reported. Furthermore, only one case of malignancy was reported, in which an Epstein-Barr virus (EBV)-associated lymphoma developed in a child who had been treated with MTX and etanercept. This observation is preliminary due to the short observation period of this analysis to compare biologic agents used contemporaneously, thus restricting the analysis to patients treated after 2011. Notably, a significant number of malignancies have been observed in the BIKER registry since 2001 [22, 23].

The observed discontinuation rate was comparable to that reported in an Italian cohort, in which 165 of 301 patients with various categories of JIA discontinued biologic treatment [24]. The majority (135 patients) discontinued for reasons of treatment failure, including a lack or loss of efficacy, and AE. Most patients discontinued due to intolerance (34.6%), which was very different compared with our cohort. This discrepancy can be explained, in part, by the frequent use of infliximab in the Italian cohort. Among this cohort, 39% of patients discontinued biologic treatment due to adverse events. Because infliximab is not approved for JIA in Germany, it cannot by studied systematically in BIKER.

In the present analysis, the overall safety of the biologic agents was acceptable. The rate of serious or medically important infections (4.1–9.5/100 patient-years) was comparable to that observed in other registry cohorts. In a British cohort, medically important infections occurred more frequently among users of TNF inhibitors than in a non-biologic control cohort, but the rate of serious infections was not significantly different [25]. Interestingly, new onset Crohn’s disease or ulcerative colitis mostly occurred in the etanercept cohort, which is consistent with previous findings [26]. Etanercept has been shown to lack efficacy toward CED [27]. However, the rate of uveitis was highest in the adalimumab cohort, which could be attributed to a selection bias, as described previously [28]. Patients with uveitis as a comorbidity had a four times higher chance of receiving adalimumab rather than etanercept [28]. Other AESI were reported infrequently. It is interesting to note that the rates of reported neutropenia, hyperlipidemia or elevated liver enzymes were not higher in the tocilizumab cohort, as might be expected based on analyses conducted in adult patients with rheumatoid arthritis [27].

The results from randomized controlled trials (RCT) cannot easily be extrapolated to routine care. Several specific features of the RCT must be considered. The inclusion and exclusion criteria may be responsible for a more homogenous study population; for example, comorbidities and concomitant drugs are usually exclusion criteria. Clinical control is tighter, prescribing practices are more stringent in a trial over time, and drug adherence may be influenced by the desire to retain a patient in the study. Thus, the RCT population may not reflect routine clinical care. By comparison, registry analyses reflect routine care and may, in part, be superior to RCTs despite having other limitations.

Observational studies of cohorts of unselected patients receiving routine care may allow better comparisons of the drugs used, although the lack of randomization must be considered when interpreting the results. In a smaller Dutch observational study of 214 patients with JIA [29], the use of etanercept and adalimumab was evaluated in routine care; however, neither the efficacy nor the survival rates were compared.

Our results reflect clinical practice and do not include the very early introduction of biologic agents, which has been evaluated in the TREAT and ACUTE-JIA studies [30, 31]. Furthermore, the observations obtained from a registry are not a substitute for clinical trials. Comparative head-to-head studies with biologic agents in juvenile idiopathic arthritis as performed in adult patients with rheumatoid arthritis would be an ideal approach, but the lower prevalence of JIA may render this an unrealistic target.

## Conclusions

So far, in clinical practice etanercept remains the most frequently used first-line biologic agent for the treatment of polyarticular JIA. The three biologic agents adalimumab, etanercept and tocilizumab had comparable efficacy. Overall, tolerance was acceptable. Interestingly, compliance was highest with tocilizumab and lowest with adalimumab. This study provides the first indication for the comparison of different biologic agents in polyarticular JIA based on observational study data, with all their weaknesses, and demonstrates the need for well-controlled head-to-head studies for confirmation.

## Additional files

Additional file 1: Table S1. Patient characteristics (unweighted as reported in BIKER). (DOCX 17 kb)
Additional file 2: Figure S1. Pediatric ACR30/50/70/90 improvement in patients receiving etanercept, adalimumab or tocilizumab as a first-line or second-line biologic agent. (PPT 172 kb)
Additional file 3: Figure S2. Drug survival during treatment with etanercept, adalimumab or tocilizumab (combined cohorts) depending on the concomitant use of methotrexate, weighted Kaplan-Meier analyses weighted by an inverse probability of treatment estimated by a generalized propensity score. No significant differences were found between the two groups. (PPT 74 kb)

## Abbreviations

AE: adverse event
AESI: adverse events of special interest
BIKER: Biologics in Pediatric Rheumatology Registry
CHAQ: Childhood Health Assessment Questionnaire
DMARD: disease-modifying antirheumatic drug
ERA: enthesitis-related arthritis
GEE: generalised estimation equations
HR: hazard ratio
IL: interleukin
JADAS: Juvenile Disease Activity Score
JIA: juvenile idiopathic arthritis
MDA: minimal disease activity
MTX: methotrexate
PedACR: Pediatric ACR criteria
PsA: psoriatic arthritis
RCT: randomized controlled trials
SAE: serious adverse event
SD: standard deviation
sJIA: systemic juvenile idiopathic arthritis
TNF: tumor necrosis factor
