# Supplementary material for: Comparison of treatment response, remission rate and drug adherence in polyarticular juvenile idiopathic arthritis patients treated with etanercept, adalimumab or tocilizumab
Source: Arthritis Res Ther. 2016 Nov 24;18:272. doi: 10.1186/s13075-016-1170-3 (PMC5122012; doi:10.1186/s13075-016-1170-3)
Supplement: Additional file 1: Table S1. — Patient characteristics (unweighted as reported in BIKER). (DOCX 17 kb) [file 13075_2016_1170_MOESM1_ESM.docx]

**Additional file 1: Table S1: Patient characteristics (unweighted as reported in BiKeR)**

|  |  |  |  |  |  |  |
| --- | --- | --- | --- | --- | --- | --- |
|  |  |  |  |  |  |  |
|  | **Etanercept cohort** | **Adalimumab cohort** | **Tocilizumab cohort** | **Adalimumab versus Etanercept** | **Tocilizumab versus Etanercept** | **Adalimumab versus Tocilizumab** |
|  | **N=419** | **N=236** | **N=74** | **OR (95%CI); p value** | **OR (95%CI); p value** | **OR (95%CI); p value** |
|  |  |  |  |  |  |  |
| Female, n (%) | 332 (79.2%) | 192 (81.4%) | 51 (68.8%) | 1.14 (0.76 ; 1.71); 0.52 | 0.66 (0.34; 1.31); 0.24 | 1.31 (0.64 ; 2.70); 0.46 |
|  |  |  |  |  |  |  |
| Age at baseline, mean +/- SD | 10.5 +/-4.4 | 11.8 +/-4.0 | 12.9+/-3.6 | 1.32 (0.66 ; 1.98); <0.001 | 2.50 (1.58 ; 3.43); <0.001 | 0.85 (0.47 ; 4.76); 0.02 |
| Median (IQR) | 11.1 (7.1-13.9) | 12.7 (8.7-15.0) | 13.5 (11.2-15.9) |  |  |  |
|  |  |  |  |  |  |  |
| Disease duration at treatment start, mean +/- SD | 3.6 +/- 3.3 | 5.8 +/- 4.0 | 6.1 +/- 3.5 | 2.19 (1.62 ; 2.76); <0.001 | 2.44 (1.56 ; 3.32); <0.001 | 0.25 (-0.68 ; 1.18); 0.600 |
| Median (IQR) | 2.6 (1.1-5.1) | 4.9 (2.4-8.4) | 5.8 (2.9-8.8) |  |  |  |
| JIA Category n (%) |  |  |  |  |  |  |
| RF+PA | 37 (8.8%) | 23 (9.7%) | 9 (12.2%) | 1.09 (0.62 ; 1.91); 0.77 | 1.06 (0.75-1.49); 0.73 | 1.07 (0.46 ; 2.47); 0.88 |
| RF-PA | 224 (53.5%) | 128 (54.2%) | 47 (63.5%) | (ref) | (ref) | (ref) |
| ExOA | 158 (37.7%) | 85 (36.0%) | 18 (24.3%) | 0.86 (0.39 ; 1.92); 0.72 | 0.54 (0.30 ; 0.97); 0.04 | 0.58 (0.31 ; 1.06); 0.08 |
|  |  |  |  |  |  |  |
| First biologic used | 400 (95.5%) | 110 (46.6%) | 14 (18.9%) | 0.04 (0.02 ; 0.07); <0.001 | 0.01 (0.01 ; 0.02); <0.001 | 0.27 (0.14 ; 0.50); <0.001 |
|  |  |  |  |  |  |  |
| Co-Med MTX, n (%) | 302 (72.1) | 127 (53.8) | 34 (45.9) | 0.45 (0.32 ; 0.63); <0.001 | 0.33 (0.20 ; 0.55); <0.001 | 0.73 (0.43 ; 1.23); 0.24 |
|  |  |  |  |  |  |  |
| JADAS10 [0-40], mean +/-SD | 13.8+/-7.1 | 12.1+/-7.6 | 15.1+/-7.4 | -1.80 (-3.14 ; -0.45); 0.01 | 1.27 (-0.95 ; 3.50); 0.26 | 3.07 (0.70 ; 5.44); 0.01 |
| Median (IQR) | 13.6 (8.8-19.0) | 11.7 (6.1-17.5) | 14.8 (9.2-20.1) |  |  |  |
|  |  |  |  |  |  |  |
| CHAQ-DI [0-3], mean +/-SD | 0.59+/-0.60 | 0.43+/-0.58 | 0.63+/-0.55 | -0.16 (-0.26 ; -0.05); <0.001 | 0.04 (-0.13 ; 0.21); 0.64 | 0.20 (0.02 ; 0.37); 0.03 |
| Median (IQR) | 0.38 (0.13-0.88) | 0.13 (0-0.623) | 0.63 (0.19-1.0) |  |  |  |
|  |  |  |  |  |  |  |
| Uveitis before start of biologic | 23 (5.5%) | 59 (25%) | 0 | 5.73 (3.43-5.99); <0.001 | - | - |
| beta = regression coefficient for continuous variables; CI = confidence interval; OR = odds ratio for categorical variable; (ref) = reference group | | | | | | |
