# Supplementary figures and images for: Comparison of treatment response, remission rate and drug adherence in polyarticular juvenile idiopathic arthritis patients treated with etanercept, adalimumab or tocilizumab
Source: Arthritis Res Ther. 2016 Nov 24;18:272. doi: 10.1186/s13075-016-1170-3 (PMC5122012; doi:10.1186/s13075-016-1170-3)

## Slide 1
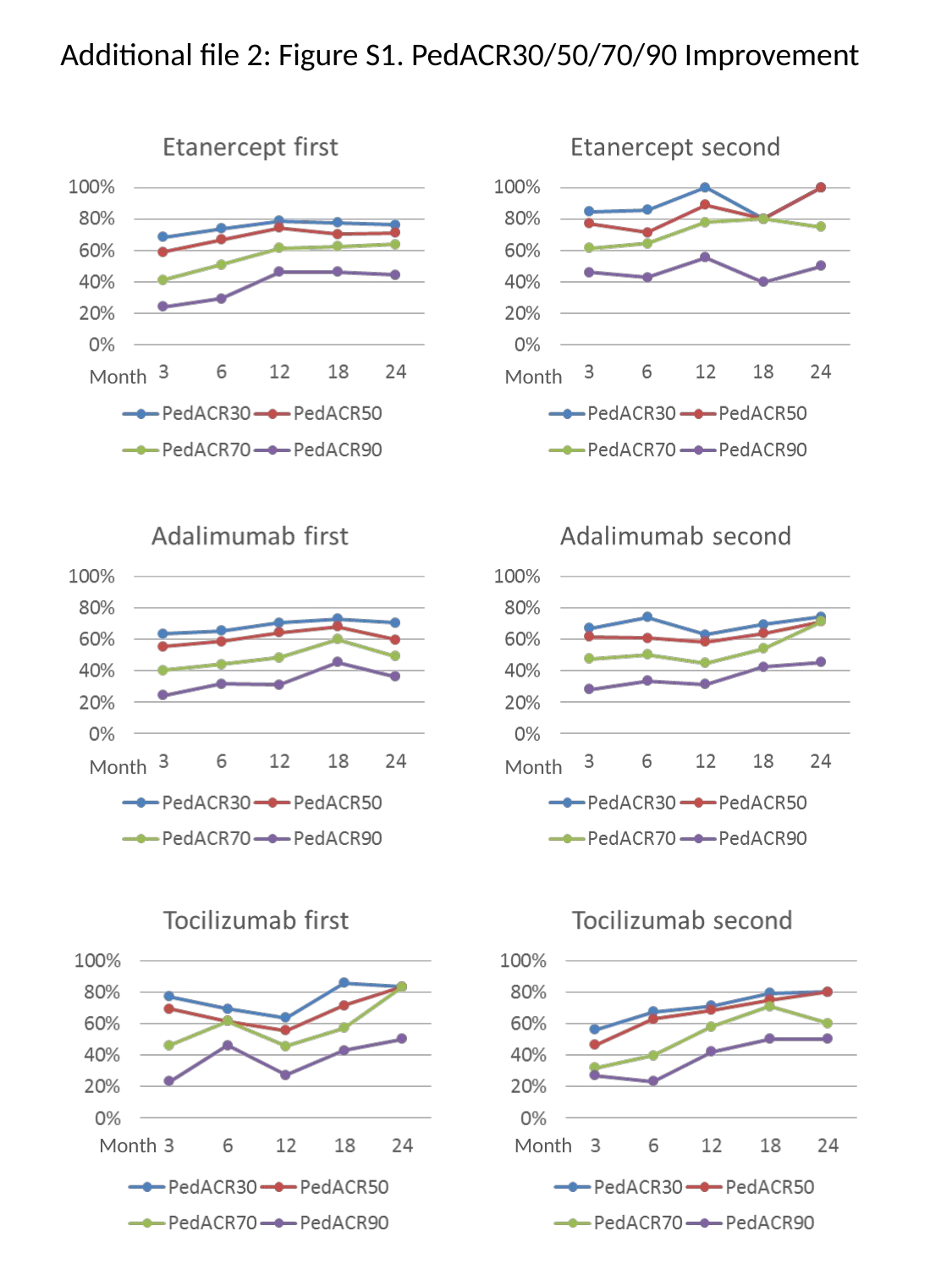

Additional file 2: Figure S1. PedACR30/50/70/90 Improvement
Month
Month
Month
Month
Month
Month

Supplement: Additional file 2: Figure S1. — Pediatric ACR30/50/70/90 improvement in patients receiving etanercept, adalimumab or tocilizumab as a first-line or second-line biologic agent. (PPT 172 kb) [file 13075_2016_1170_MOESM2_ESM.ppt]

## Slide 1
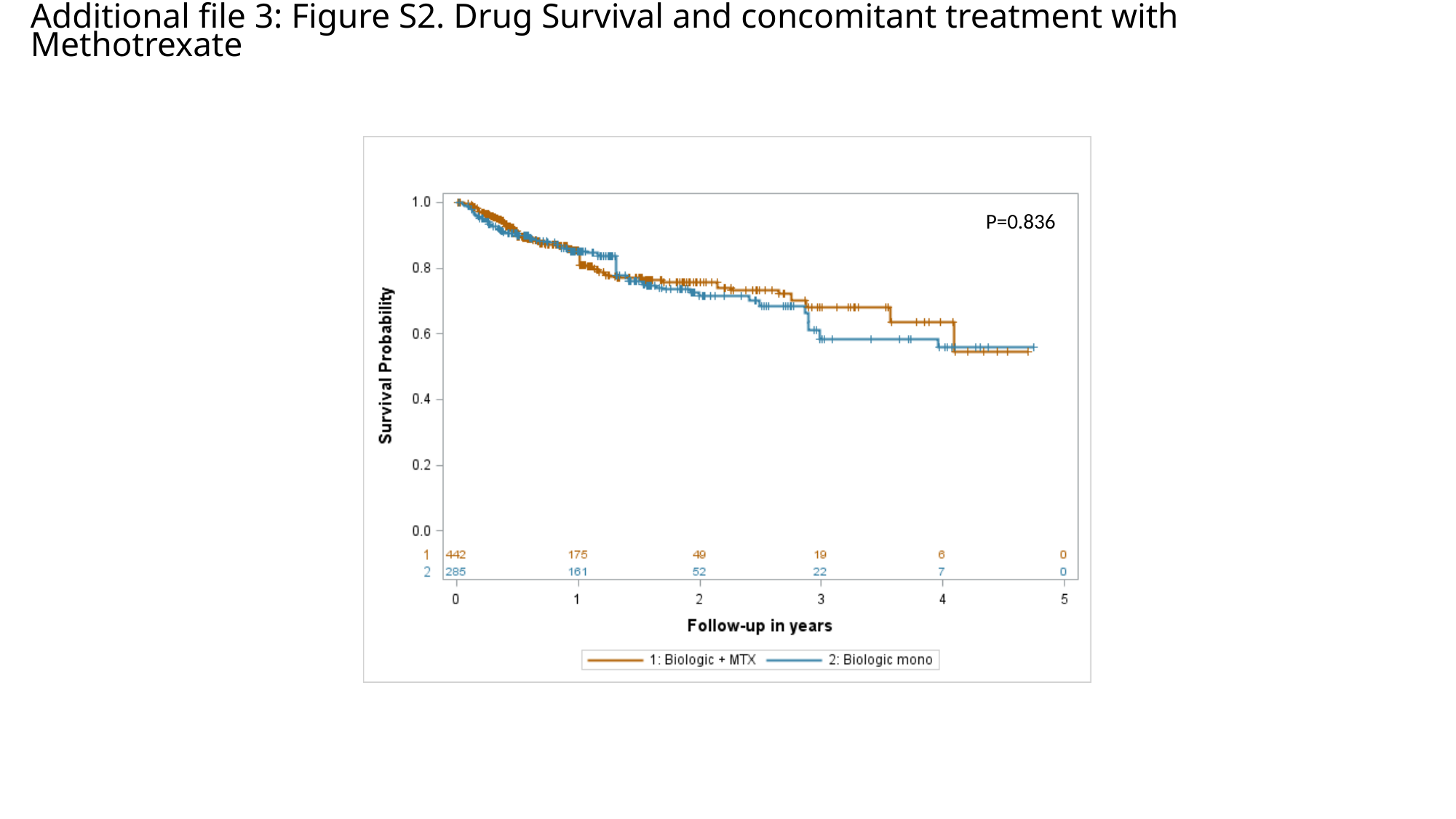

Additional file 3: Figure S2. Drug Survival and concomitant treatment with Methotrexate
P=0.836

Supplement: Additional file 3: Figure S2. — Drug survival during treatment with etanercept, adalimumab or tocilizumab (combined cohorts) depending on the concomitant use of methotrexate, weighted Kaplan-Meier analyses weighted by an inverse probability of treatment estimated by a generalized propensity score. No significant differences were found between the two groups. (PPT 74 kb) [file 13075_2016_1170_MOESM3_ESM.ppt]
